# ADULT-LIFE OCCUPATIONAL EXPOSURES: ENRICHED ENVIRONMENT OR A STRESSOR FOR THE AGING BRAIN?

**DOI:** 10.1093/geroni/igz038.094

**Published:** 2019-11-08

**Authors:** Agnieszka Z Burzynska

**Affiliations:** Colorado State University, Fort Collins, Colorado, United States

## Abstract

The positive association between midlife occupational complexity and cognitive functioning in older age is well documented. Much less is known about the underlying neural mechanisms and whether occupational stress may accelerate neurocognitive aging. According to the BOSS model (Brain aging: Occupational Stimulation and Stress), occupational exposures may serve as long-term protective and risk factors that influence the rate and extent of neurocognitive decline in aging (Burzynska, Jiao & Ganster 2018). We present findings from three independent samples linking different occupational exposures (e.g. work physical demands, innovation, autonomy, employer control) with brain volume in cognitively healthy older adults. We discuss the findings in the context of cognitive reserve and brain maintenance. Our findings suggest that occupational activities need to be acknowledged as an important factor in lifespan cognitive and brain development and warrant further research, with a possible outcome of workplace interventions aimed at optimizing neurocognitive aging.

